# Co-LncRNA: investigating the lncRNA combinatorial effects in GO annotations and KEGG pathways based on human RNA-Seq data

**DOI:** 10.1093/database/bav082

**Published:** 2015-09-10

**Authors:** Zheng Zhao, Jing Bai, Aiwei Wu, Yuan Wang, Jinwen Zhang, Zishan Wang, Yongsheng Li, Juan Xu, Xia Li

**Affiliations:** College of Bioinformatics Science and Technology, Harbin Medical University, Harbin 150081, China

## Abstract

Long non-coding RNAs (lncRNAs) are emerging as key regulators of diverse biological processes and diseases. However, the combinatorial effects of these molecules in a specific biological function are poorly understood. Identifying co-expressed protein-coding genes of lncRNAs would provide ample insight into lncRNA functions. To facilitate such an effort, we have developed Co-LncRNA, which is a web-based computational tool that allows users to identify GO annotations and KEGG pathways that may be affected by co-expressed protein-coding genes of a single or multiple lncRNAs. LncRNA co-expressed protein-coding genes were first identified in publicly available human RNA-Seq datasets, including 241 datasets across 6560 total individuals representing 28 tissue types/cell lines. Then, the lncRNA combinatorial effects in a given GO annotations or KEGG pathways are taken into account by the simultaneous analysis of multiple lncRNAs in user-selected individual or multiple datasets, which is realized by enrichment analysis. In addition, this software provides a graphical overview of pathways that are modulated by lncRNAs, as well as a specific tool to display the relevant networks between lncRNAs and their co-expressed protein-coding genes. Co-LncRNA also supports users in uploading their own lncRNA and protein-coding gene expression profiles to investigate the lncRNA combinatorial effects. It will be continuously updated with more human RNA-Seq datasets on an annual basis. Taken together, Co-LncRNA provides a web-based application for investigating lncRNA combinatorial effects, which could shed light on their biological roles and could be a valuable resource for this community.

**Database URL: **http://www.bio-bigdata.com/Co-LncRNA/

## Introduction

Large numbers of long non-coding RNAs (lncRNAs) with little or no protein-coding potential have been identified in mammalian genomes (1–3). In particular, the emergence of high-throughput RNA-Seq technology provides an unprecedented opportunity to perform comprehensive identification and characterization of lncRNAs in mammals (4, 5). Moreover, lncRNAs are known to be involved in many important biological processes, including imprinting control, cell differentiation and development, and human complex diseases (6–8). However, most lncRNAs have not been functionally characterized, and their combinatorial effects are not known with respect to a specific biological function. Therefore, systematically investigating the functions of individual or multiple lncRNAs would be an important step towards unravelling their biological roles and emphasizing the significance of this group of lncRNAs in a variety of systems and diseases.

To help researchers better understand lncRNAs and their functions, the ‘lncRNAdb’ database focuses on collecting the validated functions of lncRNAs based on the published literature (9). However, only 280 lncRNAs have been included in this database to date. Increasing evidence shows that lncRNAs can interact with DNA, RNA, protein molecules and/or in combinations, acting as essential regulators (10, 11). Several databases have made a substantial effort to collect these different types of interactions, such as RCSB databank (12), starBase (13) and lncRNAtor (14), providing protein–lncRNA interactions. However, to date there have been few interactions assayed. In turn, based on the hypothesis that lncRNA target genes are differentially expressed after lncRNA knockdown or overexpression, LncRNA2Target was developed, and a limited number of human lncRNAs were included (15).

Systematically predicting the lncRNA function has always been one of the major challenges. Functionally related genes that are involved in the same biological pathways are often regulated by similar gene regulators (16, 17). Thus, an alternative approach to genome-wide inferences to the potential function of lncRNAs is to determine whether their expression patterns correlate with those of known genes with certain functions based on co-expression analysis. For example, Ramos *et al.* (18) constructed co- expression networks, including both mRNAs and lncRNAs, to associate specific lncRNAs with specific neural cell types *in vivo* and in neurological disease states. Currently, the transcriptome data that contain both lncRNA and protein-coding genes are rapidly increasing due to the dramatic advances in RNA-Seq techniques, and several methods or databases have also been developed to fill this gap. For example, based on the re-annotated microarray data, Liao *et al.* (19) developed a tool called ‘ncFANs’, which performs functional annotation of human and mouse lncRNAs based on coding–non-coding gene co-expression networks or condition-related differentially expressed lncRNAs. Based on expression correlations between lncRNAs and protein-coding genes across 19 human normal tissues, LncRNA2Function was developed by using hypergeometric tests to functionally annotate lncRNAs with significantly enriched functional terms among the protein-coding genes that are co-expressed with the lncRNAs across 19 human normal tissues (20). In addition, ‘lncRNAtor’ developed a module to investigate only individual lncRNA function based on co-expressed protein-coding genes using collected RNA-Seq data (14). However, increasing evidence has shown that both essential biological functions and complex diseases could be affected by several lncRNAs and that the lncRNAs often function in highly complex regulatory networks (21–23). Thus, in systematic studies in which lncRNA combinatorial effects may alter a specific biological function, it is important to understand the mechanisms of complex regulations in humans; however, none of the databases has been addressed this issue.

Here, based on the re-use of 241 independent RNA-Seq datasets, we introduced Co-LncRNA, a web-based computational tool that performs enrichment analyses of expression-related genes with individual or multiple lncRNAs in all known GO annotations and KEGG pathways. The combinatorial effects of lncRNAs in the modulation of a specific biological function are investigated by our tool through the simultaneous analysis of multiple lncRNAs. Co-LncRNA offers graphical output to overview the parts of the pathways that are modulated by lncRNAs, and provides a specific tool to display relevant lncRNA–protein-coding gene co-expression networks. Co-LncRNA also allows users to upload lncRNA and protein-coding gene expression profiles to investigate the lncRNAs combinatorial effects in their relevant biological context. All of the lncRNA/protein-coding gene expression and corresponding co-expression analysis results can be downloaded freely.

## Materials and methods

### Data curation and reprocessing

**Figure 1.**
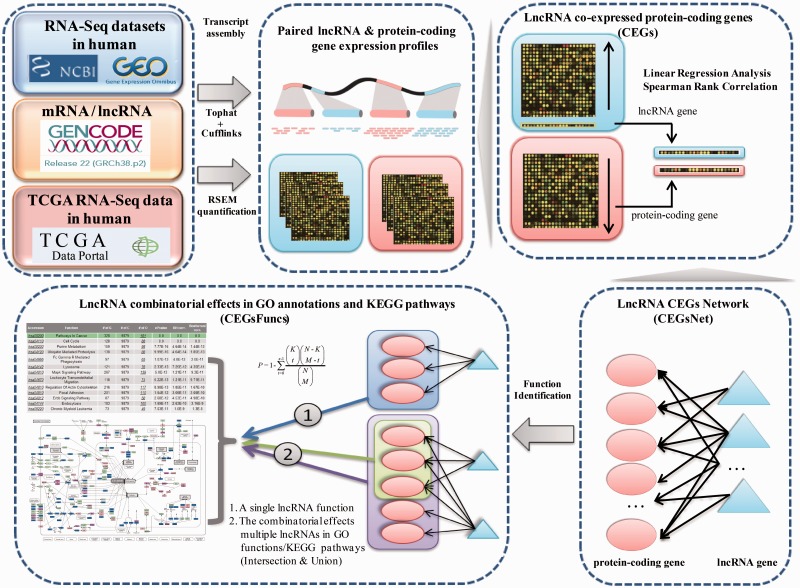
Flowchart used in Co-LncRNA for investigating the combinatorial effects of lncRNAs in GO annotations and KEGG pathways.

We followed the principle and workflow shown in Figure 1 to generate lncRNA combinatorial effects in all known GO annotations and KEGG pathways.

**Figure 2.**
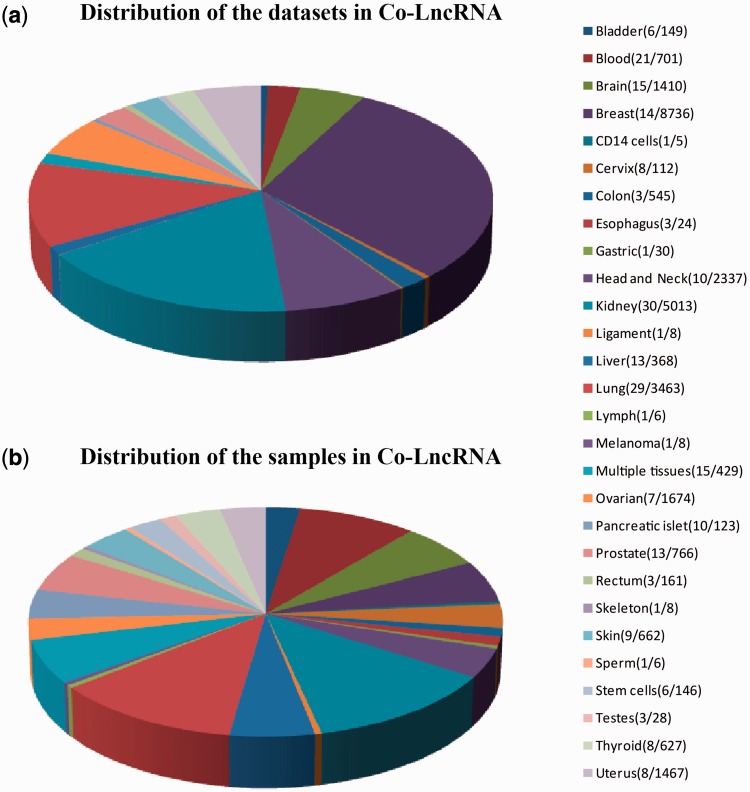
Statistics of datasets and samples used in Co-LncRNA. Distribution of (**a**) the datasets and (**b**) the samples. The two numbers behind the tissue type/cell line names represent the dataset sizes and sample sizes, respectively.

First, we curated the human total/poly(A)+ RNA-Seq datasets that are available from GEO (24) before December 2014 by searching keyword combinations of ‘human’ and ‘RNA-Seq’. Some RNA-Seq datasets from TCGA were downloaded at level 3 of the read counts to gain further insight into the combinatorial effects of lncRNAs in human cancer, which is publicly available at http://www/cancergenome.nih.gov/. For each dataset, we manually collected many types of sample information, such as tissue types/cell lines, cancer status, molecular treatment and smoker/non-smoker status. Considering the different types of samples that were mixed in one dataset, we also classified them into refined sub-groups based on the sample information that is abstracted above. All of the RNA-Seq datasets with <5 samples were excluded. In total, we obtained 241 independent datasets across 28 human tissue types/cell lines for a total of 6560 individuals (29 012 samples) (Figure 2, Table S1).

To obtain genome-wide lncRNA and protein-coding gene expression profiles from GEO datasets, we downloaded the raw data of the majority of the datasets and subsequently performed transcriptome assemblies by applying TopHat v2.0.9 and Cufflinks v2.1.1 with the default parameters (25, 26). For the other GEO datasets, their ready-made profiles were directly applied. To identify the co-expressed protein-coding genes for the lncRNAs, only the expressed genes were considered, and the threshold of the expression value was set to 0.001. For the TCGA datasets, expression profiles were obtained from RSEM quantification (27). In this study, human lncRNA and protein-coding gene annotation was directly downloaded from GENCODE v22 (28) (http://www.gencode genes.org/releases/22.html). All of the categories in the ‘Long non-coding RNA gene annotation’ GTF file were considered lncRNAs. In addition, protein-coding gene annotation was obtained from the ‘protein_coding’ category.

Next, based on reuse of the RNA-Seq sub-dataset that contained both lncRNA and protein-coding genes, we used two different methods to estimate the co-expression relationships between the lncRNA and protein-coding genes, namely, the linear regression model and Spearman rank correlation (Supplementary Methods). Moreover, the significance *P*-value of the regression/correlation coefficient was estimated. Finally, a set of co-expressed genes (CEGs) of each lncRNA were identified under a given coefficient threshold and/or threshold value of significance; in other words, the expression of these protein-coding genes was significantly associated with the lncRNA expression level. Each dataset was analysed independently.

The aforementioned regression/correlation analysis was used to identify CEGs of individual lncRNA under a specific dataset. For a given lncRNA list (two or more lncRNAs), their CEGs sets were integrated together (intersection or union of CEGs from individual dataset). Instead of performing analyses on a dataset-by-dataset basis, the CEGs of lncRNAs could also be integrated to decrease the influence of the datasets (intersection or union of the CEGs from different datasets). Then, enrichment analysis was used to detect the lncRNA combinatorial effects, and the significant *P*-values were calculated by a hypergeometric distribution (Supplementary Methods). Two kinds of correction methods, Bonferroni and Benjamini & Hochberg, were offered for multiple hypothesis testing. Two types of function information were considered, including the three branches of GO and KEGG pathways. At a given threshold of significance for the *P*-values, we could obtain the combinatorial effects of lncRNAs in all known GO annotations and KEGG pathways. Different thresholds of significance are considered here.

### Implementation

Co-LncRNA is based on a relational schema, which is supported by future Co-LncRNA updates (Figure S1). This web site was developed in JSP using a Servlet framework and is deployed on a Tomcat 6.0.33 web server, which runs under a CentOS 5.5 system. The JQuery was used to render, generate and manipulate the gene expression distribution views. The ‘CEGsNet’ module can be visualized by a Cytoscape Web tool that is embedded into Co-LncRNA. The ‘Analyse your data’ module is realized by R and Perl script. Co-LncRNA was fully tested in Google Chrome (version 17 and later).

## Results

### Database content

**Figure 3.**
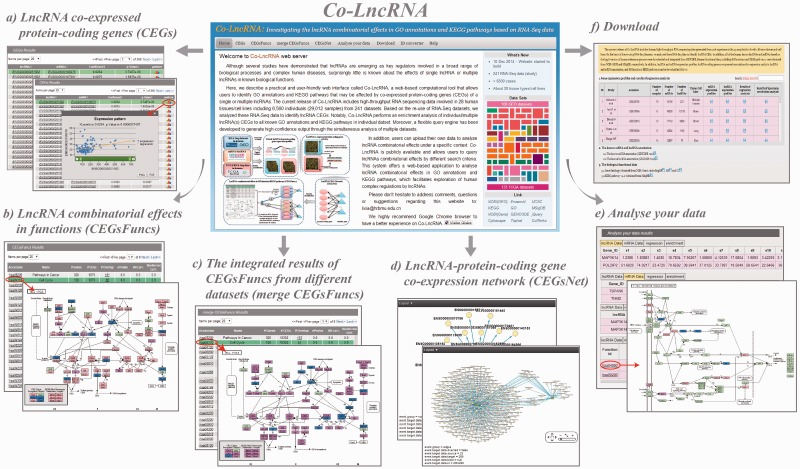
Six main modules of Co-LncRNA. (**a**) The ‘CEGs’ module provides the co-expressed associations between the lncRNA and protein-coding genes. (**b**) The ‘CEGsFuncs’ module provides the combinatorial effects of the lncRNAs in the GO annotations and KEGG pathways. (**c**) The ‘merge CEGsFuncs’ module provides the integrated results for the combinatorial effects of lncRNAs in multiple datasets. (**d**) In the ‘CEGsNet’ module, the relevant networks that were built with lncRNAs and CEGs can be visualized. (**e**) In the ‘Analyse your data’ module, the users can submit their own matched lncRNA and protein-coding gene expression profiles to investigate the combinatorial effects among the lncRNAs. (**f**) All of the internal lncRNA and protein-coding gene expression data and the lncRNA-CEG pairs can be downloaded in the ‘Download’ module for further analyses.

Co-LncRNA was designed to investigate the lncRNA combinatorial effects in the GO annotations and KEGG pathways based on human RNA-Seq data, and is available at http:// www. bio- bigdata. com/ Co- LncRNA/ . Co-LncRNA currently contains 241 independent RNA-Seq datasets across 28 human tissue types/cell lines for a total of 6560 individuals (29 012 samples) (Figure 2). Detailed information of each dataset is available in the sources provided in Supplementary Table S1. Co-LncRNA currently contains six modules (Figure 3): (a) the acquisition of lncRNA co-expressed protein-coding genes (entitled ‘CEGs’); (b) the lncRNA combinatorial effects in functions, including GO annotations and KEGG pathways under a specific dataset (entitled ‘CEGsFuncs’); (c) the integrated results of lncRNA combinatorial effects from different datasets (entitled ‘merge CEGsFuncs’); (d) the visualization of lncRNA–protein-coding gene co-expression network (entitled ‘CEGsNet’); (e) the online analysis tool (entitled ‘Analyse your data’); and (f) the download module.

After choosing the tissue/disease condition of interest and entering a gene name (lncRNA or protein-coding gene), the ‘CEGs’ module provides a list of the co- expressed pairs between the lncRNAs and protein-coding genes, as well as the values of correlation and significance (Figure 3a). In the current version of Co-LncRNA, the gene names could be represented by Ensembl gene ID or symbol, and two different types of methods were used to detect the co-expression pairs. Moreover, the users are allowed to further filter the co-expressed list by the stricter correlation or significance thresholds. For each correlated pair of lncRNA and protein-coding genes, a correlation plot was also provided in a pop-up window.

As one of the most important modules of Co-LncRNA, the ‘CEGsFuncs’ component can be used to investigate the lncRNA combinatorial effects in the GO annotations and KEGG pathways in a given dataset (Figure 3b). Similarly, the users must choose a biological condition of interest and input a single lncRNA or set of lncRNAs, and the ‘CEGsFuncs’ returns the enriched GO annotations and KEGG pathways list under a given threshold value of significance. The combinatorial effects of multiple lncRNAs are considered to be based on the union or intersection of their co-expressed protein-coding genes. The enrichment *P*-value of the union co-expressed protein-coding genes in a specific GO annotations and KEGG pathways will reflect the coordinated correlation of the function by all lncRNAs, whereas the intersection of co-expressed protein-coding genes gives an overview of the cooperative correlation of single genes by all of the lncRNAs. Moreover, co-expressed protein-coding genes that are implicated in a given pathway are graphically annotated as an overlay of the pathway wiring diagram that is provided by the KEGG database to facilitate the identification of genes of interest directly on the pathway map.

The ‘merge CEGsFuncs’ module was developed to decrease the effects of different datasets on the co-expressed protein-coding genes of lncRNAs (Figure 3c). Similar to the ‘CEGsFuncs’ module, the ‘merge CEGsFuncs’ module allows users to select multiple datasets. First, the unions or intersections of the CEGs of multiple lncRNAs within each dataset are merged. Then, the second type of merge is performed across different datasets, and it generates the integrative CEGs of the lncRNAs, which are further used to perform function enrichment analysis.

In addition, another three modules with different useful functions were provided. For a given lncRNA set, the ‘CEGsNet’ component is developed to visualize the co- expression network between these lncRNAs and corresponding co-expressed protein-coding genes, which is realized by the Cytoscape Web tool (Figure 3d). We also encouraged users to submit their own matched expression profiles of both lncRNA and protein-coding genes to the ‘Analyse your data’ module and investigate the lncRNA combinatorial effects in all known GO annotations and KEGG pathways that involve their selected biological condition (Figure 3e). Finally, all of the internal lncRNA and protein-coding gene expression data and corresponding co-expression relationships can be freely downloaded in the ‘Download’ component for further analyses (Figure 3f).

### Case study

Co-LncRNA allows users to investigate the function effects of individual lncRNA or multiple lncRNAs in known biological functions under user-selected tissue(s) or disease(s) context. Take a combination of MALAT1 with TUG1 as an example. Several studies suggested that high MALAT1 expression is associated with poor prognosis in non-small cell lung cancer (NSCLC), whose elevated expression is also associated with cellular hyper-proliferation (29, 30). Zhang *et al.* (31) reported that lncRNA TUG1 can regulate cell proliferation in NSCLC. To infer putative combinational functional effects for these two lncRNAs in lung cancer, we first separately obtained the CEGs of MALAT1 and TUG1 in the ‘CEGs’ module by using the ‘Lung (GEO Seo JS *et al*.)’ dataset, which contains 87 lung adenocarcinomas and 77 adjacent normal tissues. The correlation method was set to the linear regression model, and the threshold of regression significance was 0.01. As a result, there are 7191 CEGs for MALAT1 and 8087 CEGs for TUG1. Next, in the ‘CEGsFuncs’ module, we performed an enrichment analysis of integrated CEGs (union of CEGs) of these two lncRNAs in the KEGG pathways by using the ‘Lung (GEO Seo JS *et al*.)’ dataset. In agreement with previous reports, the ‘CEGsFuncs’ results showed that these two lncRNAs can significantly affect the ‘KEGG pathways in cancer’, ‘Cell Cycle’ and other various pathways (*P* < 0.05) (Figure 3b). Moreover, to decrease the effects of different datasets, we further integrated two datasets (a union of datasets), the ‘Lung (GEO Seo JS *et al*.)’ and ‘Lung (GEO Kim SC *et al.*)’ datasets, to investigate the combinatorial effects of lncRNAs in multiple datasets in the ‘merge CEGsFuncs’ module. Similarly, these two lncRNAs are also involved in ‘KEGG pathways in cancer’ and ‘Cell Cycle’ (Figure 3c). These results indicate that lncRNAs can cooperate with another to modulate a specific biological function.

## Conclusions and future directions

To the best of our knowledge, Co-LncRNA provides a series of highly specific tools for lncRNA-related analysis, including identifying lncRNA co-expressed protein-coding genes, and investigating single lncRNA or lncRNA combination effects in GO annotations and KEGG pathways. We hope that Co-LncRNA will become an efficient tool that is easy to use and that can be incorporated successfully into lncRNA-related analysis pipelines, particularly for lncRNA-targeted functional analysis. Considering the heterogeneity between different datasets, the subsequent analysis of RNA-Seq data will also be appropriately adjusted following each dataset’s specifications. The system will be continuously updated with more human RNA-Seq datasets on an annual basis. As the number of human RNA-Seq experiments increases, after both manual curation and computational analysis, we will incorporate them into Co-LncRNA.

## Supplementary Data

Supplementary data are available at *Database* Online.

## Funding

National High Technology Research and Development Program of China (863 Program, 2014AA021102); National Program on Key Basic Research Project (973 Program, 2014CB910504); National Natural Science Foundation of China Fund (91439117, 61473106 and 61203264); China Postdoctoral Science Foundation (2014T70364, 2015M571436 and LBH-Z14134); Natural Science Foundation of Heilongjiang Province (QC2015020); WeihanYu Youth Science Fund Project of Harbin Medical University; Harbin Special Funds of Innovative Talents on Science and Technology Research Project (RC2015QN003080); Innovation Research Fund for Graduate Students of Harbin Medical University (YJSCX2014-22HYD). Funding for open access charge: National High Technology Research and Development Program of China (863 Program, 2014AA021102); National Program on Key Basic Research Project (973 Program, 2014CB910504); National Natural Science Foundation of China Fund (91439117, 61473106 and 61203264).

*Conflict of interest.* None declared.

## Supplementary Material

Supplementary Data
