# Supplementary material for: Co-LncRNA: investigating the lncRNA combinatorial effects in GO annotations and KEGG pathways based on human RNA-Seq data
Source: Database (Oxford). 2015 Sep 10;2015:bav082. doi: 10.1093/database/bav082 (PMC4565967; doi:10.1093/database/bav082)
Supplement: Supplementary Data [file supp_2015_bav082_index.html]

Co-LncRNA: investigating the lncRNA combinatorial effects in GO annotations and KEGG pathways based on human RNA-Seq data — Supplementary Data 

# Co-LncRNA: investigating the lncRNA combinatorial effects in GO annotations and KEGG pathways based on human RNA-Seq data

## Supplementary Data

files

- Supplementary Data - doc file
