# Supplementary material for: Co-LncRNA: investigating the lncRNA combinatorial effects in GO annotations and KEGG pathways based on human RNA-Seq data
Source: Database (Oxford). 2015 Sep 10;2015:bav082. doi: 10.1093/database/bav082 (PMC4565967; doi:10.1093/database/bav082)
Supplement: Supplementary Data [file supp_bav082_Supplementary.doc]

**
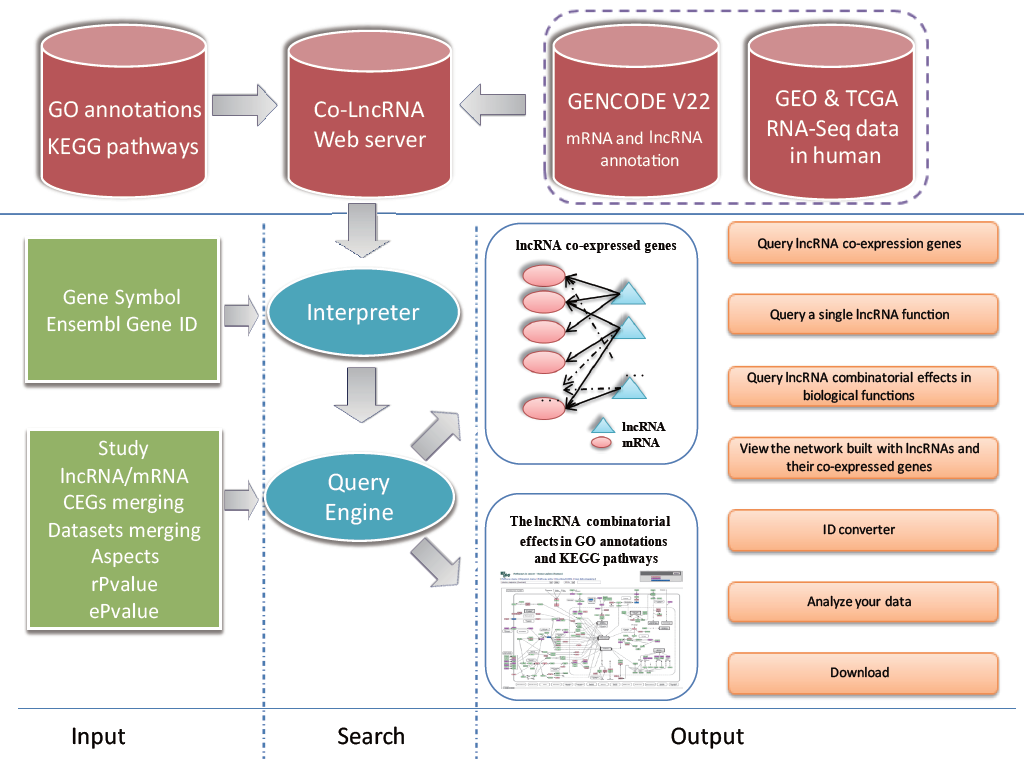
**

**Supplementary Figure 1**

**Overview of building and workflow of Co-LncRNA.** Co-LncRNA aims to provide a web-based computational tool to investigate the lncRNA combinatorial effects in GO annotations and KEGG pathways based on human RNA-Seq data. Users can input a set of lncRNAs of interest to identify their combination action in user-selected individual or multiple datasets. The analysis tools enable users to carry out online analysis based on re-using human RNA-Seq data, including identification of co-expressed relationships of lncRNA genes with protein-coding genes, lncRNA combinatorial effects in biological functions under individual dataset, recurrent lncRNA combinatorial effects under multiple datasets, visualization of lncRNA-protein-coding co-expression network and user own data online analysis. All lncRNA/protein-coding gene expression profiles and their co-expression analysis results can be downloaded freely.

**Supplementary Table 1**

**Data details about RNA-Seq datasets in Co-LncRNA.**

| **source** | **tissue/cell line** | **title** | **description** | **sample sizes** | **lncRNA gene size** | **protein-coding gene size** |
| --- | --- | --- | --- | --- | --- | --- |
| **133 TCGA datasets** | | | | | | |
| TCGA | Bladder | Bladder (TCGA BLUCA normal vs tumor) | Bladder urothelial carcinoma: Normal vs Tumor | 45 | 935 | 14739 |
| TCGA | Bladder | Bladder (TCGA BLUCA pathologic stage) | Bladder urothelial carcinoma: Pathologic stage | 33 | 929 | 14711 |
| TCGA | Bladder | Bladder (TCGA BLUCA person neoplasm) | Bladder urothelial carcinoma: Person neoplasm cancer status | 31 | 929 | 14700 |
| TCGA | Brain | Brain (TCGA BLGG drug Temodar) | Brain lower grade glioma: Drug Temodar or not | 84 | 952 | 14821 |
| TCGA | Brain | Brain (TCGA BLGG drug Temozolomide) | Brain lower grade glioma: Drug Temozolomide or not | 84 | 952 | 14821 |
| TCGA | Brain | Brain (TCGA BLGG histological type) | Brain lower grade glioma: Histological type | 205 | 954 | 14825 |
| TCGA | Brain | Brain (TCGA BLGG neoplasm histologic grade) | Brain lower grade glioma: Neoplasm histologic grade | 205 | 954 | 14825 |
| TCGA | Brain | Brain (TCGA BLGG person neoplasm) | Brain lower grade glioma: Person neoplasm cancer status | 163 | 952 | 14822 |
| TCGA | Brain | Brain (TCGA BLGG therapy CHE) | Brain lower grade glioma: Combination of Chemotherapy and Hormone therapy | 83 | 952 | 14821 |
| TCGA | Brain | Brain (TCGA GBM drug Temodar) | Glioblastoma multiforme: Drug Temodar or not | 125 | 1163 | 15659 |
| TCGA | Brain | Brain (TCGA GBM drug Temozolomide) | Glioblastoma multiforme: Drug Temozolomide or not | 125 | 1163 | 15659 |
| TCGA | Brain | Brain (TCGA GBM person neoplasm) | Glioblastoma multiforme: Person neoplasm cancer status | 149 | 1165 | 15674 |
| TCGA | Brain | Brain (TCGA GBM therapy CHE) | Glioblastoma multiforme: Combination of Chemotherapy and Hormone therapy | 125 | 1163 | 15659 |
| TCGA | Breast | Breast (TCGA BRICA axillary lymph node) | Breast invasive carcinoma: Axillary lymph node stage (method type) | 605 | 952 | 14963 |
| TCGA | Breast | Breast (TCGA BRICA drug Arimidex) | Breast invasive carcinoma: Drug Arimidex or not | 325 | 951 | 14961 |
| TCGA | Breast | Breast (TCGA BRICA drug CycDox) | Breast invasive carcinoma: Drug Combination of Cyclophosphamide and Doxorubicin | 325 | 951 | 14961 |
| TCGA | Breast | Breast (TCGA BRICA drug Doxorubicin) | Breast invasive carcinoma: Drug Doxorubicin | 325 | 951 | 14961 |
| TCGA | Breast | Breast (TCGA BRICA estrogen receptor) | Breast invasive carcinoma: Breast carcinoma estrogen receptor status | 754 | 952 | 14963 |
| TCGA | Breast | Breast (TCGA BRICA Her2/neu receptor) | Breast invasive carcinoma: HER2/neu immunohistochemistry receptor status | 669 | 951 | 14964 |
| TCGA | Breast | Breast (TCGA BRICA histological type) | Breast invasive carcinoma: Histological type | 780 | 952 | 14963 |
| TCGA | Breast | Breast (TCGA BRICA normal vs tumor) | Breast invasive carcinoma: Normal vs Tumor | 894 | 953 | 14966 |
| TCGA | Breast | Breast (TCGA BRICA pathologic M) | Breast invasive carcinoma: Pathologic M | 788 | 952 | 14964 |
| TCGA | Breast | Breast (TCGA BRICA pathologic N) | Breast invasive carcinoma: Pathologic N | 785 | 952 | 14963 |
| TCGA | Breast | Breast (TCGA BRICA pathologic stage) | Breast invasive carcinoma: Pathologic stage | 788 | 952 | 14964 |
| TCGA | Breast | Breast (TCGA BRICA person neoplasm) | Breast invasive carcinoma: Person neoplasm cancer status | 619 | 952 | 14960 |
| TCGA | Breast | Breast (TCGA BRICA progesterone receptor) | Breast invasive carcinoma: Progesterone receptor status | 751 | 952 | 14961 |
| TCGA | Breast | Breast (TCGA BRICA therapy CHE) | Breast invasive carcinoma: Combination of Chemotherapy and Hormone therapy | 328 | 951 | 14961 |
| TCGA | Cervix | Cervix (TCGA CESC drug Cisplatin) | Cervical squamous cell carcinoma and endocervical adenocarcinoma: Drug Cisplatin or not | 12 | 825 | 14916 |
| TCGA | Cervix | Cervix (TCGA CESC histological type) | Cervical squamous cell carcinoma and endocervical adenocarcinoma: Neoplasm histologic grade | 23 | 828 | 14932 |
| TCGA | Cervix | Cervix (TCGA CESC pathologic M) | Cervical squamous cell carcinoma and endocervical adenocarcinoma: Pathologic M | 20 | 829 | 14930 |
| TCGA | Cervix | Cervix (TCGA CESC pathologic N) | Cervical squamous cell carcinoma and endocervical adenocarcinoma: Pathologic N | 20 | 829 | 14930 |
| TCGA | Cervix | Cervix (TCGA CESC person neoplasm) | Cervical squamous cell carcinoma and endocervical adenocarcinoma: Person neoplasm cancer status | 20 | 825 | 14933 |
| TCGA | Colon | Colon (TCGA COAD person neoplasm) | Colon adenocarcinoma: Person neoplasm cancer status | 186 | 802 | 13975 |
| TCGA | Colon | Colon (TCGA COAD residual tumor) | Colon adenocarcinoma: Residual tumor | 177 | 802 | 13971 |
| TCGA | Colon | Colon (TCGA COAD tumor stage) | Colon adenocarcinoma: Tumor stage | 182 | 802 | 13977 |
| TCGA | Head and Neck | Head and Neck (TCGA HNSC anatomic neoplasm) | Head and Neck squamous cell carcinoma: Anatomic neoplasm subdivision | 298 | 858 | 14755 |
| TCGA | Head and Neck | Head and Neck (TCGA HNSC clinical stage) | Head and Neck squamous cell carcinoma: Cinical stage | 302 | 858 | 14755 |
| TCGA | Head and Neck | Head and Neck (TCGA HNSC drug Carboplatin) | Head and Neck squamous cell carcinoma: Drug Carboplatin or not | 91 | 855 | 14747 |
| TCGA | Head and Neck | Head and Neck (TCGA HNSC drug CisCar) | Head and Neck squamous cell carcinoma: Drug Combination of Cisplatin and Carboplatin | 91 | 855 | 14747 |
| TCGA | Head and Neck | Head and Neck (TCGA HNSC drug Cisplatin) | Head and Neck squamous cell carcinoma: Drug Cisplatin | 91 | 855 | 14747 |
| TCGA | Head and Neck | Head and Neck (TCGA HNSC neoplasm histologic grade) | Head and Neck squamous cell carcinoma: Neoplasm histologic grade | 302 | 858 | 14755 |
| TCGA | Head and Neck | Head and Neck (TCGA HNSC normal vs tumor) | Head and Neck squamous cell carcinoma: Normal vs Tumor | 341 | 859 | 14758 |
| TCGA | Head and Neck | Head and Neck (TCGA HNSC pathologic N) | Head and Neck squamous cell carcinoma: Pathologic N | 294 | 859 | 14756 |
| TCGA | Head and Neck | Head and Neck (TCGA HNSC pathologic stage) | Head and Neck squamous cell carcinoma: Pathologic stage | 257 | 859 | 14760 |
| TCGA | Head and Neck | Head and Neck (TCGA HNSC person neoplasm) | Head and Neck squamous cell carcinoma: Person neoplasm cancer status | 270 | 860 | 14755 |
| TCGA | Kidney | Kidney (TCGA KICH hemoglobin result) | Kidney chromophobe: Hemoglobin result | 23 | 861 | 14399 |
| TCGA | Kidney | Kidney (TCGA KICH normal vs tumor) | Kidney chromophobe: Normal vs Tumor | 91 | 864 | 14434 |
| TCGA | Kidney | Kidney (TCGA KICH pathologic stage) | Kidney chromophobe: Pathologic stage | 23 | 861 | 14397 |
| TCGA | Kidney | Kidney (TCGA KICH person neoplasm) | Kidney chromophobe: Person neoplasm cancer status | 24 | 862 | 14397 |
| TCGA | Kidney | Kidney (TCGA KICH serum calcium result) | Kidney chromophobe: Serum calcium result | 19 | 861 | 14398 |
| TCGA | Kidney | Kidney (TCGA KICH white cell count result) | Kidney chromophobe: White cell count result | 23 | 861 | 14399 |
| TCGA | Kidney | Kidney (TCGA KIRC drug Sorafenib) | Kidney renal clear cell carcinoma: Drug Sorafenib or not | 80 | 927 | 14337 |
| TCGA | Kidney | Kidney (TCGA KIRC hemoglobin result) | Kidney renal clear cell carcinoma: Hemoglobin result | 414 | 928 | 14342 |
| TCGA | Kidney | Kidney (TCGA KIRC neoplasm histologic grade) | Kidney renal clear cell carcinoma: Neoplasm histologic grade | 480 | 928 | 14343 |
| TCGA | Kidney | Kidney (TCGA KIRC normal vs tumor) | Kidney renal clear cell carcinoma: Normal vs Tumor | 552 | 929 | 14345 |
| TCGA | Kidney | Kidney (TCGA KIRC pathologic M) | Kidney renal clear cell carcinoma: Pathologic M | 481 | 928 | 14343 |
| TCGA | Kidney | Kidney (TCGA KIRC pathologic N) | Kidney renal clear cell carcinoma: Pathologic N | 481 | 928 | 14343 |
| TCGA | Kidney | Kidney (TCGA KIRC pathologic stage) | Kidney renal clear cell carcinoma: Pathologic stage | 481 | 928 | 14343 |
| TCGA | Kidney | Kidney (TCGA KIRC person neoplasm) | Kidney renal clear cell carcinoma: Person neoplasm cancer status | 455 | 928 | 14342 |
| TCGA | Kidney | Kidney (TCGA KIRC serum calcium result) | Kidney renal clear cell carcinoma: Serum calcium result | 328 | 928 | 14340 |
| TCGA | Kidney | Kidney (TCGA KIRC serum calcium result) | Kidney renal clear cell carcinoma: White cell count result | 52 | 978 | 14858 |
| TCGA | Kidney | Kidney (TCGA KIRC white cell count result) | Kidney renal papillary cell carcinoma: Hemoglobin result | 403 | 927 | 14341 |
| TCGA | Kidney | Kidney (TCGA KIRP hemoglobin result) | Kidney renal papillary cell carcinoma: Normal vs Tumor | 66 | 978 | 14861 |
| TCGA | Kidney | Kidney (TCGA KIRP normal vs tumor) | Kidney renal papillary cell carcinoma: Pathologic M | 101 | 976 | 14864 |
| TCGA | Kidney | Kidney (TCGA KIRP pathologic M) | Kidney renal papillary cell carcinoma: Pathologic N | 69 | 977 | 14861 |
| TCGA | Kidney | Kidney (TCGA KIRP pathologic N) | Kidney renal papillary cell carcinoma: Pathologic stage | 75 | 978 | 14862 |
| TCGA | Kidney | Kidney (TCGA KIRP pathologic stage) | Kidney renal papillary cell carcinoma: Person neoplasm cancer status | 67 | 977 | 14862 |
| TCGA | Kidney | Kidney (TCGA KIRP person neoplasm) | Kidney renal papillary cell carcinoma: Serum calcium result | 71 | 978 | 14860 |
| TCGA | Kidney | Kidney (TCGA KIRP white cell count result) | Kidney renal papillary cell carcinoma: White cell count result | 66 | 978 | 14861 |
| TCGA | Liver | Liver (TCGA LIHC inflammation extent type) | Liver hepatocellular carcinoma: Adjacent hepatic tissue inflammation extent type | 23 | 914 | 14729 |
| TCGA | Liver | Liver (TCGA LIHC invasion type) | Liver hepatocellular carcinoma: Vascular tumor cell invasion type | 34 | 922 | 14772 |
| TCGA | Liver | Liver (TCGA LIHC neoplasm histologic grade) | Liver hepatocellular carcinoma: Neoplasm histologic grade | 38 | 922 | 14770 |
| TCGA | Liver | Liver (TCGA LIHC normal vs tumor) | Liver hepatocellular carcinoma: Normal vs Tumor | 65 | 917 | 14760 |
| TCGA | Liver | Liver (TCGA LIHC pathologic M) | Liver hepatocellular carcinoma: Pathologic M | 38 | 921 | 14772 |
| TCGA | Liver | Liver (TCGA LIHC pathologic N) | Liver hepatocellular carcinoma: Pathologic N | 37 | 922 | 14772 |
| TCGA | Liver | Liver (TCGA LIHC pathologic stage) | Liver hepatocellular carcinoma: Pathologic stage | 29 | 924 | 14777 |
| TCGA | Liver | Liver (TCGA LIHC person neoplasm) | Liver hepatocellular carcinoma: Person neoplasm cancer status | 32 | 922 | 14775 |
| TCGA | Liver | Liver (TCGA LIHC residual tumor) | Liver hepatocellular carcinoma: Residual tumor | 36 | 921 | 14772 |
| TCGA | Lung | Lung (TCGA LUAD anatomic neoplasm) | Lung adenocarcinoma: Anatomic neoplasm subdivision | 231 | 1065 | 15419 |
| TCGA | Lung | Lung (TCGA LUAD drug carboplatin) | Lung adenocarcinoma: Drug Carboplatin or not | 59 | 1066 | 15415 |
| TCGA | Lung | Lung (TCGA LUAD drug cisplatin) | Lung adenocarcinoma: Drug Cisplatin | 59 | 1066 | 15415 |
| TCGA | Lung | Lung (TCGA LUAD drug CisVin) | Lung adenocarcinoma: Drug Combination of Cisplatin and Vinorelbine | 31 | 1066 | 15415 |
| TCGA | Lung | Lung (TCGA LUAD histological type) | Lung adenocarcinoma: Histological type | 216 | 1064 | 15418 |
| TCGA | Lung | Lung (TCGA LUAD normal vs tumor) | Lung adenocarcinoma: Normal vs Tumor | 291 | 1067 | 15421 |
| TCGA | Lung | Lung (TCGA LUAD pathologic M) | Lung adenocarcinoma: Pathologic M | 232 | 1065 | 15419 |
| TCGA | Lung | Lung (TCGA LUAD pathologic N) | Lung adenocarcinoma: Pathologic N | 232 | 1065 | 15419 |
| TCGA | Lung | Lung (TCGA LUAD pathologic stage) | Lung adenocarcinoma: Pathologic stage | 231 | 1064 | 15419 |
| TCGA | Lung | Lung (TCGA LUAD person neoplasm) | Lung adenocarcinoma: Person neoplasm cancer status | 166 | 1065 | 15418 |
| TCGA | Lung | Lung (TCGA LUSC drug carboplatin) | Lung squamous cell carcinoma: Drug Carboplatin or not | 46 | 864 | 14925 |
| TCGA | Lung | Lung (TCGA LUSC drug CarTax) | Lung squamous cell carcinoma: Drug Combination of Carboplatin and Taxol | 44 | 863 | 14924 |
| TCGA | Lung | Lung (TCGA LUSC normal vs tumor) | Lung squamous cell carcinoma: Normal vs Tumor | 391 | 865 | 14935 |
| TCGA | Lung | Lung (TCGA LUSC person neoplasm) | Lung squamous cell carcinoma: Person neoplasm cancer status | 208 | 867 | 14933 |
| TCGA | Lung | Lung (TCGA LUSC residual tumor) | Lung squamous cell carcinoma: Residual tumor | 277 | 867 | 14932 |
| TCGA | Lung | Lung (TCGA LUSC tumor stage) | Lung squamous cell carcinoma: Tumor stage | 311 | 867 | 14933 |
| TCGA | Ovarian | Ovarian (TCGA OV drug carboplatin) | Ovarian serous cystadenocarcinoma: Drug Carboplatin | 237 | 995 | 14951 |
| TCGA | Ovarian | Ovarian (TCGA OV drug CarPan) | Ovarian serous cystadenocarcinoma: Drug Combination of Carboplatin and Panclitaxel | 227 | 995 | 14951 |
| TCGA | Ovarian | Ovarian (TCGA OV drug CarTax) | Ovarian serous cystadenocarcinoma: Drug Combination of Carboplatin and Taxol | 215 | 995 | 14951 |
| TCGA | Ovarian | Ovarian (TCGA OV neoplasm histologic grade) | Ovarian serous cystadenocarcinoma: Neoplasm histologic grade | 258 | 995 | 14951 |
| TCGA | Ovarian | Ovarian (TCGA OV person neoplasm) | Ovarian serous cystadenocarcinoma: Person neoplasm cancer status | 234 | 995 | 14948 |
| TCGA | Ovarian | Ovarian (TCGA OV therapy CHE) | Ovarian serous cystadenocarcinoma: Combination of Chemotherapy and Hormone therapy | 239 | 995 | 14951 |
| TCGA | Ovarian | Ovarian (TCGA OV tumor stage) | Ovarian serous cystadenocarcinoma: Tumor stage | 264 | 995 | 14950 |
| TCGA | Pancreatic islet | Pancreatic islet (TCGA PAAD neoplasm histologic grade) | Pancreatic adenocarcinoma: Neoplasm histologic grade | 14 | 927 | 15117 |
| TCGA | Pancreatic islet | Pancreatic islet (TCGA PAAD pathologic M) | Pancreatic adenocarcinoma: Pathologic M | 17 | 927 | 15118 |
| TCGA | Pancreatic islet | Pancreatic islet (TCGA PAAD pathologic N) | Pancreatic adenocarcinoma: Pathologic N | 18 | 929 | 15120 |
| TCGA | Pancreatic islet | Pancreatic islet (TCGA PAAD pathologic stage) | Pancreatic adenocarcinoma: Pathologic stage | 13 | 927 | 15117 |
| TCGA | Pancreatic islet | Pancreatic islet (TCGA PAAD person neoplasm) | Pancreatic adenocarcinoma: Person neoplasm cancer status | 18 | 929 | 15120 |
| TCGA | Pancreatic islet | Pancreatic islet (TCGA PAAD residual tumor) | Pancreatic adenocarcinoma: Residual tumor | 17 | 928 | 15120 |
| TCGA | Prostate | Prostate (TCGA PRAD normal vs tumor) | Prostate adenocarcinoma: Normal vs Tumor | 220 | 884 | 14928 |
| TCGA | Prostate | Prostate (TCGA PRAD person neoplasm) | Prostate adenocarcinoma: Person neoplasm cancer status | 141 | 885 | 14923 |
| TCGA | Prostate | Prostate (TCGA PRAD residual tumor) | Prostate adenocarcinoma: Residual tumor | 146 | 885 | 14922 |
| TCGA | Prostate | Prostate (TCGA PRAD tumor level) | Prostate adenocarcinoma: Tumor level | 86 | 883 | 14916 |
| TCGA | Rectum | Rectum (TCGA READ drug 5-Fluorouracil) | Rectum adenocarcinoma: Drug 5-Fluorouracil or not | 21 | 924 | 15030 |
| TCGA | Rectum | Rectum (TCGA READ residual tumor) | Rectum adenocarcinoma: Residual tumor | 70 | 929 | 15044 |
| TCGA | Rectum | Rectum (TCGA READ tumor stage) | Rectum adenocarcinoma: Tumor stage | 70 | 929 | 15047 |
| TCGA | Skin | Skin (TCGA SKCM drug Interferon) | Skin cutaneous melanoma: Drug Interferon or not | 16 | 790 | 14163 |
| TCGA | Skin | Skin (TCGA SKCM pathologic N) | Skin cutaneous melanoma: Pathologic N | 180 | 791 | 14176 |
| TCGA | Skin | Skin (TCGA SKCM pathologic stage) | Skin cutaneous melanoma: Pathologic stage | 175 | 790 | 14176 |
| TCGA | Skin | Skin (TCGA SKCM person neoplasm) | Skin cutaneous melanoma: Person neoplasm cancer status | 197 | 790 | 14177 |
| TCGA | Thyroid | Thyroid (TCGA THCA histological type) | Thyroid carcinoma: Histological type | 73 | 891 | 14631 |
| TCGA | Thyroid | Thyroid (TCGA THCA normal vs tumor) | Thyroid carcinoma: Normal vs Tumor | 93 | 892 | 14636 |
| TCGA | Thyroid | Thyroid (TCGA THCA pathologic M) | Thyroid carcinoma: Pathologic M | 80 | 891 | 14633 |
| TCGA | Thyroid | Thyroid (TCGA THCA pathologic N) | Thyroid carcinoma: Pathologic N | 82 | 891 | 14633 |
| TCGA | Thyroid | Thyroid (TCGA THCA pathologic stage) | Thyroid carcinoma: Pathologic stage | 80 | 891 | 14633 |
| TCGA | Thyroid | Thyroid (TCGA THCA person neoplasm) | Thyroid carcinoma: Person neoplasm cancer status | 72 | 891 | 14627 |
| TCGA | Thyroid | Thyroid (TCGA THCA primary neoplasm) | Thyroid carcinoma: Primary neoplasm focus type | 75 | 891 | 14632 |
| TCGA | Thyroid | Thyroid (TCGA THCA residual tumor) | Thyroid carcinoma: Residual tumor | 72 | 890 | 14636 |
| TCGA | Uterus | Uterus (TCGA UCEC drug CarPan) | Uterine corpus endometrioid carcinoma: Drug Combination Carboplatin and Panclitaxel | 100 | 936 | 14982 |
| TCGA | Uterus | Uterus (TCGA UCEC drug CarTax) | Uterine corpus endometrioid carcinoma: Drug Combination of Carboplatin and Taxol | 100 | 936 | 14985 |
| TCGA | Uterus | Uterus (TCGA UCEC histological type) | Uterine corpus endometrioid carcinoma: Histological type | 301 | 930 | 14997 |
| TCGA | Uterus | Uterus (TCGA UCEC neoplasm histologic grade) | Uterine corpus endometrioid carcinoma: Neoplasm histologic grade | 308 | 929 | 14999 |
| TCGA | Uterus | Uterus (TCGA UCEC person neoplasm) | Uterine corpus endometrioid carcinoma: Person neoplasm cancer status | 283 | 929 | 14997 |
| TCGA | Uterus | Uterus (TCGA UCEC residual tumor) | Uterine corpus endometrioid carcinoma: Residual tumor | 258 | 929 | 15002 |
| TCGA | Uterus | Uterus (TCGA UCEC therapy CHE) | Uterine corpus endometrioid carcinoma: Combination of Chemotherapy and Hormone therapy | 102 | 936 | 14984 |
| **108 GEO datasets** | | | | | | |
| GEO | Bladder | Bladder (GEO Chen J *et al. a*) | GSE12946 | 16 | 5953 | 16940 |
| GEO | Bladder | Bladder (GEO Chen J *et al. b*) | GSE13652 | 6 | 6531 | 17145 |
| GEO | Bladder | Bladder (GEO Chen J *et al.*) | GSE15780 | 8 | 5664 | 15602 |
| GEO | Blood | Blood (GEO Bansal A *et al.*) | GSE16921 | 41 | 100 | 13404 |
| GEO | Blood | Blood (GEO Beyer M *et al.*) | GSE19166 | 6 | 5136 | 16721 |
| GEO | Blood | Blood (GEO Breen MS et al.) | GSE19480 | 161 | 4127 | 14771 |
| GEO | Blood | Blood (GEO Deng X *et al.*) | GSE19486 | 24 | 5909 | 15553 |
| GEO | Blood | Blood (GEO Gregor A *et al.*) | GSE19486_notTNF | 14 | 5964 | 15613 |
| GEO | Blood | Blood (GEO Hoek KL et al.) | GSE19486_TNF | 10 | 6035 | 15641 |
| GEO | Blood | Blood (GEO Kasowski et al. a) | GSE20156 | 14 | 86 | 12759 |
| GEO | Blood | Blood (GEO Kasowski et al. b) | GSE22260 | 30 | 4910 | 16751 |
| GEO | Blood | Blood (GEO Kasowski *et al.*) | GSE22260_c | 10 | 5203 | 16829 |
| GEO | Blood | Blood (GEO Li M *et al.*) | GSE22260_n | 20 | 4734 | 16701 |
| GEO | Blood | Blood (GEO Majoros WH *et al.*) | GSE23316 | 28 | 1687 | 12700 |
| GEO | Blood | Blood (GEO Mayer ML *et al.*) | GSE24283 | 8 | 7744 | 17572 |
| GEO | Blood | Blood (GEO Nguyen LS *et al.*) | GSE24952 | 20 | 6869 | 17354 |
| GEO | Blood | Blood (GEO Piazza R *et al.*) | GSE24952_kc | 10 | 7078 | 17410 |
| GEO | Blood | Blood (GEO Pickrell JK *et al.*) | GSE24952_kn | 10 | 7154 | 17400 |
| GEO | Blood | Blood (GEO Shi H et al.) | GSE25840 | 27 | 5882 | 15862 |
| GEO | Blood | Blood (GEO Slavoff SA *et al.*) | GSE26109 | 7 | 4059 | 16069 |
| GEO | Blood | Blood (GEO Sun XJ *et al.*) | GSE27199 | 6 | 43 | 9827 |
| GEO | Blood | Blood (GEO Toung JM *et al.*) | GSE28123 | 142 | 31 | 13259 |
| GEO | Blood | Blood (GEO Windhager L *et al.*) | GSE28866 | 93 | 66 | 12341 |
| GEO | Blood | Blood (GEO Yoon OK *et al.*) | GSE29006 | 8 | 650 | 11097 |
| GEO | Brain | Brain (GEO Au KF *et al.*) | GSE29155 | 11 | 3137 | 14225 |
| GEO | Brain | Brain (GEO Florio M et al.) | GSE29158 | 20 | 99 | 13509 |
| GEO | Brain | Brain (GEO Irimia M et al.) | GSE29160 | 6 | 6743 | 17262 |
| GEO | Brain | Brain (GEO Ramskold D *et al.*) | GSE30554 | 9 | 3922 | 15706 |
| GEO | Brain | Brain (GEO Voineagu I *et al.*) | GSE30573 | 6 | 5477 | 17009 |
| GEO | CD14 cells | CD14 cells (GEO Gemma C *et al.*) | GSE30611 | 16 | 5179 | 16910 |
| GEO | Cervix | Cervix (GEO Liu HW *et al.*) | GSE31486 | 6 | 4105 | 14661 |
| GEO | Cervix | Cervix (GEO Tolstorukov MY *et al.*) | GSE31614 | 34 | 6740 | 17444 |
| GEO | Cervix | Cervix (GEO Yao C et al.) | GSE31614_b | 20 | 6517 | 17213 |
| GEO | Esophagus | Esophagus (GEO Tong M *et al. a*) | GSE31614_bc | 10 | 6608 | 17267 |
| GEO | Esophagus | Esophagus (GEO Tong M *et al. b*) | GSE31614_bn | 10 | 6925 | 17280 |
| GEO | Esophagus | Esophagus (GEO Tong M *et al.*) | GSE31614_t | 14 | 7525 | 17768 |
| GEO | Gastric | Gastric (GEO Kim YH *et al.*) | GSE31614_tc | 7 | 7002 | 17566 |
| GEO | Kidney | Kidney (GEO Huelga SC *et al.*) | GSE31614_tn | 7 | 7461 | 17663 |
| GEO | Kidney | Kidney (GEO JACK LI *et al. a*) | GSE31653 | 7 | 4177 | 13808 |
| GEO | Kidney | Kidney (GEO JACK LI *et al. b*) | GSE31728 | 58 | 993 | 9798 |
| GEO | Kidney | Kidney (GEO JACK LI *et al.*) | GSE32307 | 15 | 150 | 15136 |
| GEO | Kidney | Kidney (GEO Kloster MB *et al.*) | GSE32424 | 12 | 3220 | 15439 |
| GEO | Kidney | Kidney (GEO Luo Z *et al.*) | GSE32424_en | 5 | 1978 | 13986 |
| GEO | Ligament | Ligament (GEO Rai MF et al.) | GSE32424_et | 7 | 3940 | 15813 |
| GEO | Liver | Liver (GEO Chan TH *et al.*) | GSE33154 | 12 | 3692 | 13911 |
| GEO | Liver | Liver (GEO Losic B et al.) | GSE33294 | 6 | 5353 | 16598 |
| GEO | Liver | Liver (GEO Luna JM et al.) | GSE33816 | 12 | 7180 | 16553 |
| GEO | Liver | Liver (GEO Van Delft J *et al.*) | GSE34097 | 10 | 4919 | 16373 |
| GEO | Lung | Lung (GEO Beane J *et al.*) | GSE34329 | 8 | 6040 | 16193 |
| GEO | Lung | Lung (GEO Gao X *et al.*) | GSE34740 | 9 | 2790 | 13226 |
| GEO | Lung | Lung (GEO Kim SC *et al. a*) | GSE34780 | 6 | 112 | 13315 |
| GEO | Lung | Lung (GEO Kim SC *et al. b*) | GSE34995 | 30 | 3980 | 15105 |
| GEO | Lung | Lung (GEO Kim SC *et al.*) | GSE35126 | 18 | 103 | 13031 |
| GEO | Lung | Lung (GEO Liu R et al.) | GSE35296 | 10 | 5169 | 16877 |
| GEO | Lung | Lung (GEO Raskatov JA *et al.*) | GSE35296_control | 5 | 5099 | 16793 |
| GEO | Lung | Lung (GEO Seo JS *et al. a*) | GSE35296_cytokine | 5 | 4742 | 16690 |
| GEO | Lung | Lung (GEO Seo JS *et al. b*) | GSE35394 | 17 | 2460 | 13829 |
| GEO | Lung | Lung (GEO Seo JS *et al.*) | GSE35584 | 20 | 7598 | 17063 |
| GEO | Lung | Lung (GEO Trapnell C *et al. a*) | GSE36242 | 8 | 1931 | 14016 |
| GEO | Lung | Lung (GEO Trapnell C *et al. b*) | GSE36695 | 12 | 285 | 14137 |
| GEO | Lung | Lung (GEO Trapnell C *et al.*) | GSE36952 | 6 | 126 | 13535 |
| GEO | Lymph | Lymph (GEO Shukla S *et al.*) | GSE36968 | 30 | 126 | 15496 |
| GEO | Melanoma | Melanoma (GEO Zhou Q et al.) | GSE37521 | 96 | 68 | 7931 |
| GEO | Multiple tissues | Multiple tissues (GEO Adiconis X *et al.*) | GSE37703 | 12 | 2415 | 14092 |
| GEO | Multiple tissues | Multiple tissues (GEO Brunner AL *et al.*) | GSE37703_hiseq | 6 | 3031 | 14501 |
| GEO | Multiple tissues | Multiple tissues (GEO Cabili MN *et al.*) | GSE37703_miseq | 6 | 2148 | 13853 |
| GEO | Multiple tissues | Multiple tissues (GEO Chen J *et al.*) | GSE37764 | 11 | 6094 | 17432 |
| GEO | Multiple tissues | Multiple tissues (GEO ENCODE DCC *et al.*) | GSE37764_c | 6 | 6713 | 17645 |
| GEO | Multiple tissues | Multiple tissues (GEO Farrell CM *et al.*) | GSE37764_n | 5 | 6015 | 17367 |
| GEO | Multiple tissues | Multiple tissues (GEO Gertz J *et al.*) | GSE38234 | 16 | 135 | 15153 |
| GEO | Multiple tissues | Multiple tissues (GEO Giacomini CP *et al.*) | GSE38495 | 16 | 3437 | 15395 |
| GEO | Multiple tissues | Multiple tissues (GEO Kin K et al.) | GSE38770 | 5 | 7910 | 17179 |
| GEO | Multiple tissues | Multiple tissues (GEO Myers R *et al.*) | GSE39170 | 15 | 7687 | 18164 |
| GEO | Multiple tissues | Multiple tissues (GEO Pan Q *et al.*) | GSE39661 | 17 | 32 | 14319 |
| GEO | Multiple tissues | Multiple tissues (GEO Raz T *et al.*) | GSE39821 | 7 | 6016 | 16788 |
| GEO | Multiple tissues | Multiple tissues (GEO Sendler E *et al.*) | GSE40050 | 12 | 5634 | 15674 |
| GEO | Multiple tissues | Multiple tissues (GEO Wang ET *et al.*) | GSE40310 | 8 | 14 | 8331 |
| GEO | Multiple tissues | Multiple tissues (GEO Xu W *et al.*) | GSE40419 | 164 | 180 | 16000 |
| GEO | Pancreatic islet | Pancreatic islet (GEO Eizirik DL *et al. a*) | GSE40419_lc | 87 | 187 | 16044 |
| GEO | Pancreatic islet | Pancreatic islet (GEO Eizirik DL *et al. b*) | GSE40419_n | 77 | 180 | 15912 |
| GEO | Pancreatic islet | Pancreatic islet (GEO Eizirik DL *et al.*) | GSE40705 | 18 | 227 | 13052 |
| GEO | Pancreatic islet | Pancreatic islet (GEO Frietze S et al.) | GSE40859 | 6 | 5477 | 14925 |
| GEO | Prostate | Prostate (GEO Decker KF *et al.*) | GSE41264 | 6 | 45 | 10125 |
| GEO | Prostate | Prostate (GEO Kannan K *et al. a*) | GSE41745 | 6 | 5608 | 16053 |
| GEO | Prostate | Prostate (GEO Kannan K *et al. b*) | GSE42146 | 13 | 68 | 13249 |
| GEO | Prostate | Prostate (GEO Kannan K *et al.*) | GSE42326 | 5 | 4073 | 14366 |
| GEO | Prostate | Prostate (GEO Kim JH *et al.*) | GSE43834 | 10 | 259 | 14530 |
| GEO | Prostate | Prostate (GEO Li H *et al.*) | GSE44718 | 8 | 3798 | 15804 |
| GEO | Prostate | Prostate (GEO Nacu S *et al.*) | GSE45133 | 7 | 4089 | 16205 |
| GEO | Prostate | Prostate (GEO Prensner JR *et al.*) | GSE45833 | 17 | 7741 | 16261 |
| GEO | Prostate | Prostate (GEO Tewari AK *et al.*) | GSE46217 | 5 | 150 | 11084 |
| GEO | Skeleton | Skeleton (GEO Koeppel M *et al.*) | GSE46611 | 5 | 3036 | 14424 |
| GEO | Skin | Skin (GEO Berger MF *et al.*) | GSE46831 | 11 | 358 | 15573 |
| GEO | Skin | Skin (GEO Chang AL *et al.*) | GSE63420 | 14 | 5219 | 16659 |
| GEO | Skin | Skin (GEO Jabbari A *et al.*) | GSE63733 | 12 | 3770 | 14396 |
| GEO | Skin | Skin (GEO Loayza-Puch F *et al.*) | GSE63776 | 6 | 922 | 14066 |
| GEO | Skin | Skin (GEO Tsoi LC et al.) | GSE63979 | 42 | 5138 | 17044 |
| GEO | Sperm | Sperm (GEO Johnson GD *et al.*) | GSE64018 | 24 | 994 | 12774 |
| GEO | Stem cells | Stem cells (GEO Choi KD *et al.*) | GSE64098 | 40 | 481 | 14721 |
| GEO | Stem cells | Stem cells (GEO Gkountela S *et al.*) | GSE64655 | 56 | 3456 | 14211 |
| GEO | Stem cells | Stem cells (GEO Jaager K *et al.*) | GSE64677 | 8 | 64 | 12242 |
| GEO | Stem cells | Stem cells (GEO Oda H et al.) | GSE64752 | 9 | 240 | 14106 |
| GEO | Stem cells | Stem cells (GEO Peng S *et al.*) | GSE64813 | 188 | 280 | 14032 |
| GEO | Stem cells | Stem cells (GEO Qiao Y et al.) | GSE65000 | 10 | 247 | 18062 |
| GEO | Testes | Testes (GEO Chen J *et al. a*) | GSE65469 | 8 | 6852 | 16370 |
| GEO | Testes | Testes (GEO Chen J *et al. b*) | GSE66117 | 52 | 82 | 11604 |
| GEO | Testes | Testes (GEO Chen J *et al.*) | GSE66719 | 8 | 270 | 12272 |
| GEO | Uterus | Uterus (GEO Gertz J *et al.*) | GSE66777 | 5 | 291 | 13145 |

**Supplementary Methods**

## Identification of lncRNA co-expressed protein-coding genes

Based on re-using the RNA-Seq data containing both lncRNA and protein-coding genes, we used two distinct methods to estimate the co-expression relationships between the lncRNA and protein-coding genes, namely, the linear regression model and the Spearman rank correlation. For each dataset, this model was performed between a coding gene ***c*** and a lncRNA ***lnc*** in a given dataset with ***n*** samples. Each dataset was performed independently.

***The linear regression model:***

$y_{c,i}=\beta_{0}+\beta_{lnc}x_{lnc,i}+\varepsilon_{i}$, $i=1,...,n$

In this linear model, ***y_c,i_*** is the expression level of coding gene ***c*** in sample ***i***. ***x_lnc,i_*** is the expression level of lncRNA ***lnc*** in sample ***i***. **𝛽_0_** is the intercept, **𝛽*_lnc_*** is the regression coefficients for lncRNA expression variable, and **𝜀** is the error term. We use the R function ***lm(y~x)*** method to obtain an estimate for the lncRNA coefficient and the significance of the regression coefficient.

***The Spearman rank correlation:***

Replacing the R function ***cor.test (y, x, method="spearman")*** with longer runtime, we use the MATLAB function ***corr (y, x, 'type', 'Spearman')*** to estimate the correlation coefficient and its significance.

## Predicting potential functions altered by a given lncRNA list

For a given lncRNA list (two or more lncRNAs), we integrated their CEGs sets into a gene set (intersection or union of CEGs) and identified candidate functional terms by performing enrichment analysis in each biological functions, including three branches of GO annotations and KEGG pathways. Here, if a biological function is significantly enriched by the integrated gene set, it is appreciated that these lncRNAs might coordinate to be involved in this biological function. Enrichment analysis is realized by hypergeometric distribution, and the probability *P* for co-expressed genes in a given functional term is calculated according to:

$$P=1-\sum_{t=0}^{x-1} \frac{\left(\begin{matrix} K \\ t \end{matrix}\right)\left(\begin{matrix} N-K \\ M-t \end{matrix}\right)}{\left(\begin{matrix} N \\ M \end{matrix}\right)}$$

Where ***N*** is the total number of all human protein-coding genes (default background distribution), ***K*** is the total number of protein-coding genes that are annotated in the given functional term, ***M*** is the size of the integrated co-expressed gene subset, ***x*** is the number of the integrated gene set that are also annotated to the function term.
